# Supplementary material for: Neural computations underpinning the strategic management of influence in advice giving
Source: Nat Commun. 2017 Dec 19;8:2191. doi: 10.1038/s41467-017-02314-5 (PMC5736665; doi:10.1038/s41467-017-02314-5)
Supplement: Supplementary file 1 — Supplementary Information [file 41467_2017_2314_MOESM1_ESM.pdf]

Supplementary Information
Supplementary Figures

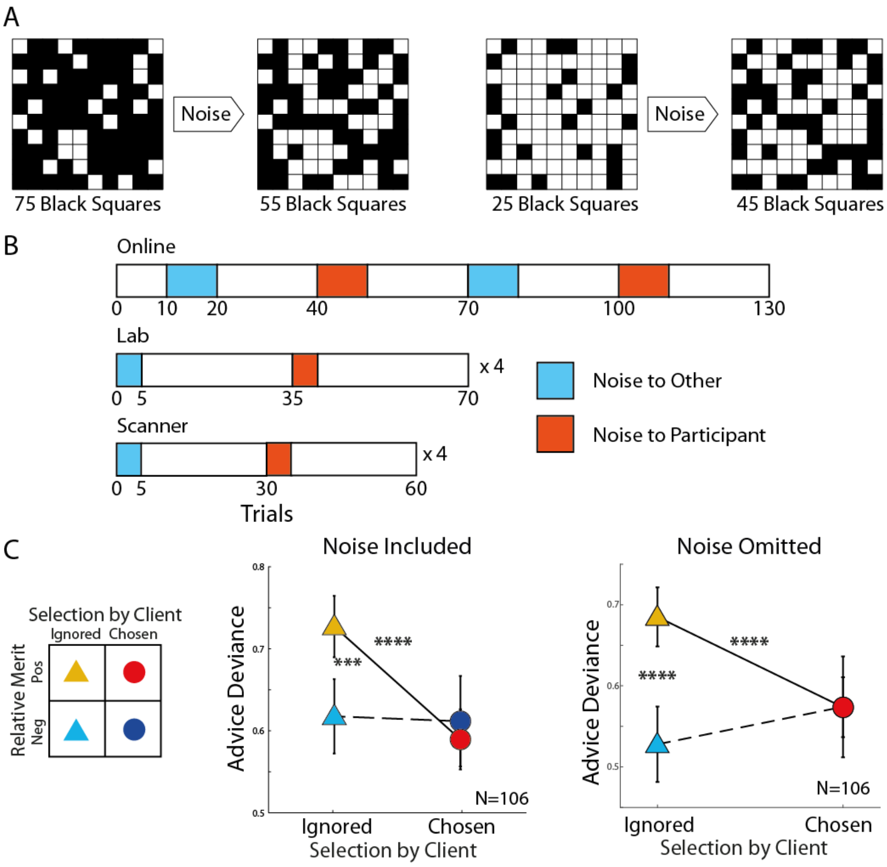

Supplementary Figure 1: Selective Manipulation of Advice Quality
We used a manipulation to increase the probability of differences in advice and accuracy between advisers, which would then entail client switching between advisers. (A) We therefore introduced noise to one of the advisers' evidence, i.e. the ratio between black and white squares in the grid. The noise procedure went as follows. If the probability of the coin being in the black urn on a specific trial was 0.75, the grid would normally include 75 black squares and 25 white squares (right side of panel A). On a noisy trial, this composition changed to 55 black squares and 45 white squares, i.e. reduction of contrast by 20 squares. Similarly, when the probability of the coin being in the *white* urn on a specific trial was 0.75 (0.25 probability of being in the black), the noisy grid would include 55 white squares (45 black squares) instead of 75 white squares (25 black squares) in the not noisy case, again reducing the contrast by 20 squares (left side of panel A). In all noisy trials' contrasts were reduced by 20 squares in a similar fashion. (B) Noisy periods were relatively short, lasting 10 consecutive trials in the online experiment and 5 consecutive trials in the lab and scanner experiments. Noise was introduced either to the participant or to the other adviser (i.e. the virtual rival algorithm was fed noisy evidence). Online experiments included 4 blocks of noisy periods, 2 for each adviser, while the longer lab based and scanner experiments included 8 blocks of noisy period, 4 for each adviser. (C) We analysed the data before and after omitting the noise periods. Our results did not change after omitting the noisy trials (compare two panels), with a significant main effect of Relative Merit ($F(1,315) = 5.29$, $p = 0.02$), and significant Relative Merit x Selection by Client effect ($F(1,315) = 13.1$, $p = 0.0005$). Error bars indicate SEM.

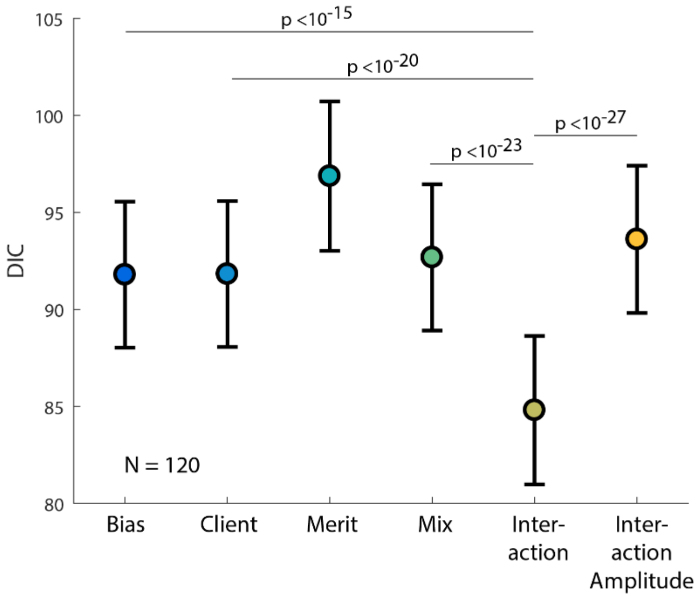

Supplementary Figure 2: Models Comparison
Mean and SEM of the models' DIC scores, as well as p values for two-sided paired t-test comparisons. We fitted our models using a Markov chain Monte Carlo (MCMC) Metropolis algorithm. This process resulted in likelihood distribution across parameter space that minimizes the individual log likelihood (likelihood of advice deviance given the model's parameter estimation). We calculated individual Deviance Information Criterion (DIC) [66], which uses the distribution of likelihood obtained and penalizes for increased number of parameters. All our models included a Bias parameter to capture trait overconfidence or under-confidence bias. Our baseline model included no other parameters (Bias model), while other models included a Selection by Client parameter (Client), a Relative Merit Parameter (Merit), both Selection by Client and a Relative Merit parameters (Mix), and an additional Interaction parameter (Interaction). An additional model was tested which was identical to the 'Interaction' model but used the magnitude and sign of relative merit instead of only the sign of relative merit (see Methods). Best fit to participants' behaviour was obtained using the Interaction model (see main text). See all estimated models parameters in table S1.

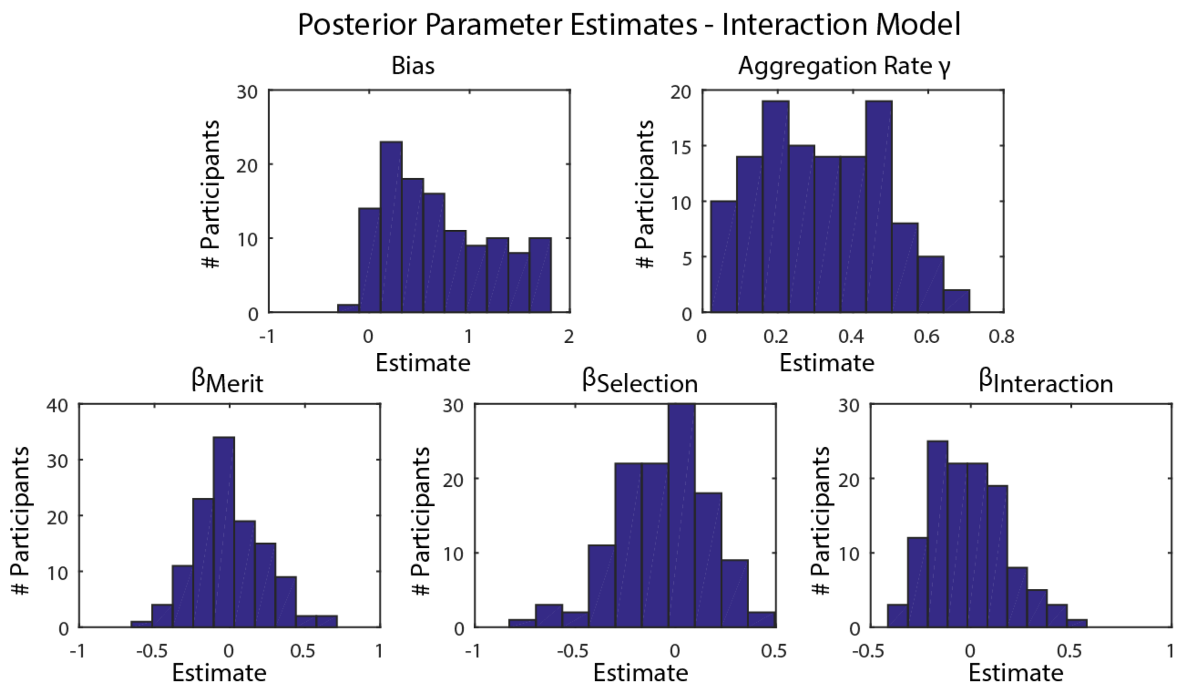

Supplementary Figure 3: Distributions of Individual Estimated Parameters from the Interaction Model

Our model fitting procedure provided distribution of parameter estimation for each participant. We calculated the posterior individual parameters of the Interaction model to examine individual differences in weights assigned to client selection, relative merit and their interaction. In all figures the histogram of posterior parameters across participants is displayed (n=120). Most of our participants were overconfident (bias > 0), and had below 0.8 aggregation rate γ. Weight assigned to relative merit was distributed around zero and not significantly different from 0. The weight assigned to client selection was variable but significantly lower than zero (mean = -0.065, p = 0.002). Weight assigned to the interaction of relative merit and selection was distributed around zero and not significantly different from 0.

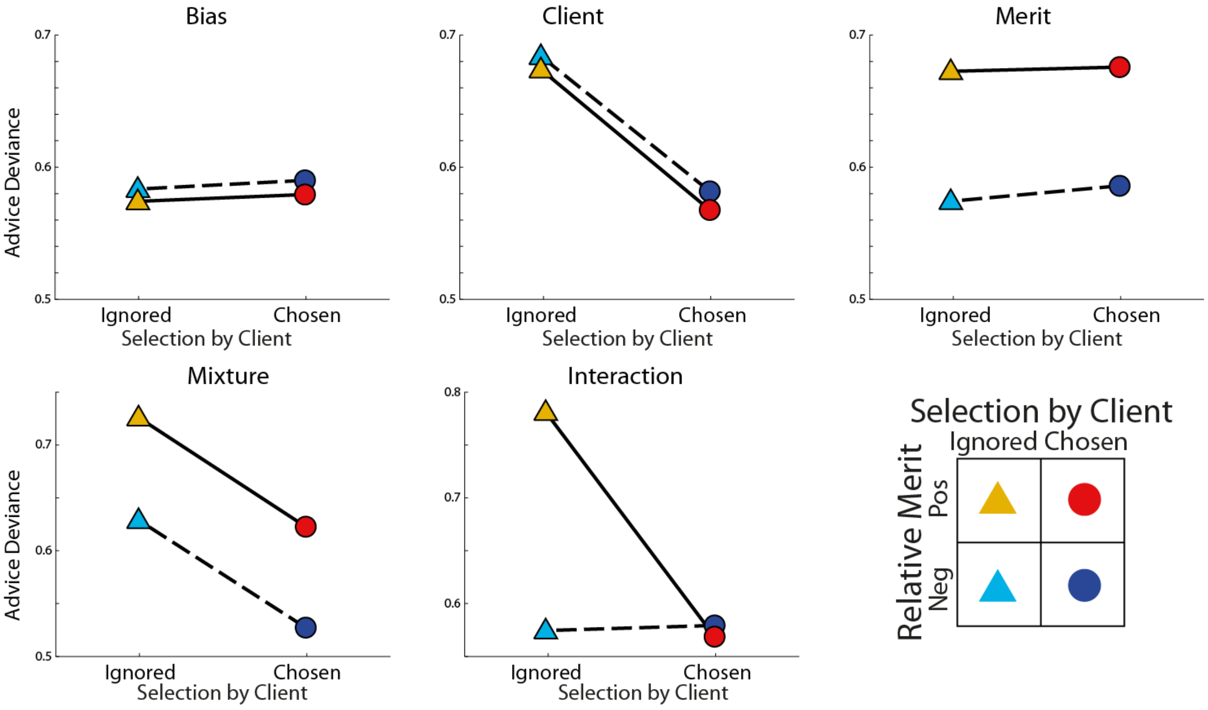

Supplementary Figure 4: Models Simulations
We ran simulations of our five models and examined how they qualitatively differ from one another, and how well they capture patterns in the data. We used fixed values for free parameters, and the other adviser advices and coin location probabilities from the real obtained data, and estimated advice deviance declared by the participants according to the different models. These simulations show that only a model that take into account client selection, relative merit and their interaction can reproduce the pattern of results observed in participants' advice deviance.

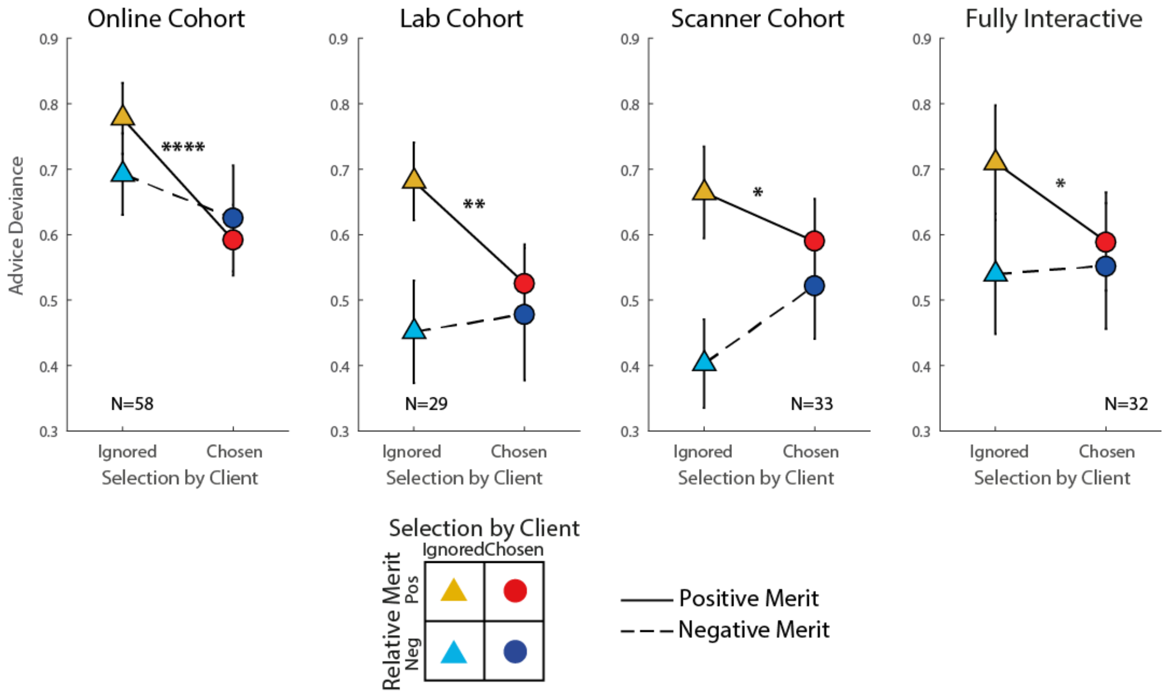

Supplementary Figure 5: Client Selection and Relative Merit Effect in the Four Cohorts
Aggregated advice deviance was analysed independently in our three cohorts of participants. The main result holds in all cohorts: advisers gave more determined advices when the client chose the rival and ignored them and their relative merit was positive, compared to when the client chose them and they had positive relative merit (two-tailed paired t-test comparisons, * $p < 0.05$, ** $p < 0.005$, **** $p < 0.00005$). Error bars indicate SEM. However, deviance in negative relative merit condition varied across cohorts. We carried a mixed effects ANOVA (2x2x4) to examine the cohort dependent variations with selection, relative merit and Cohort as main fixed effects, and participants as random effect nested in Cohort. This resulted in significant main effect for relative merit ($F(1, 412) = 24.4$, $p < 0.0001$), and main selection effect ($F(1, 452) = 6.37$, $p = 0.012$),but no Cohort effect ($F(3, 452) = 1.03$, $p = 0.34$). As expected from the repeated main result, interaction between relative merit and selection was significant ($F(1, 452) = 23.3$, $p < 0.0001$). Interaction between relative merit and Cohort was marginal ($F(3, 452) = 2.41$, $p = 0.07$), as was the interaction between selection and Cohort ($F(3, 452) = 2.34$, $p = 0.075$). The triple interaction between relative merit, selection and Cohort was not significant ($F(3, 452) = 0.36$, $p = 0.77$)). The main result of relative merit and selection interaction sustained across cohorts and experimental settings, including the fully interactive experiment.

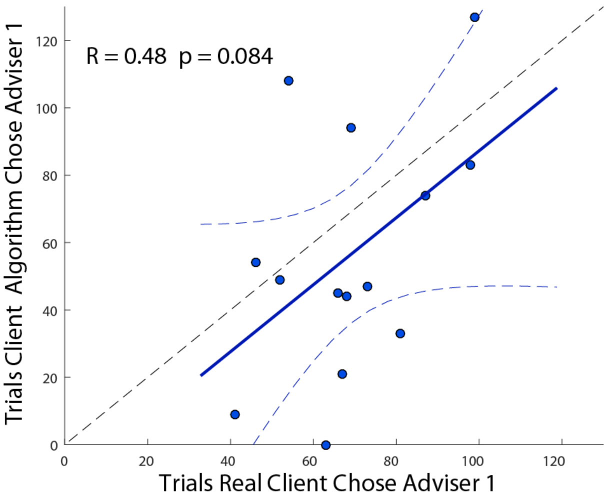

Supplementary Figure 6: Comparison of live client and algorithm behaviour in the live interaction experiment

We simulated the choices that our algorithm would have made facing the advice history of the two advisers and the outcome, and compared these simulations with the real choices made by the participants who played the client. An obvious caveat here is that N=16 clients was perhaps too small a sample size to test this hypothesis. Notwithstanding the sample size caveat, we found some correspondence between the number of trials that human and virtual clients chose adviser 1 (R = 0.48, p = 0.084). When compared directly, we did not find a significant difference between the number of times the algorithm and adviser choose adviser 1 (Mean_Algorithm = 56.28, Mean_Real_Client = 68.85, t(15) = 1.45, p = 0.17). This finding indicates that the algorithm may bare similarity to real clients, capturing some basic elements of the human clients' behaviour. Both real clients and the algorithm's choices of advisers were influenced by the adviser's history of accuracy and confidence.

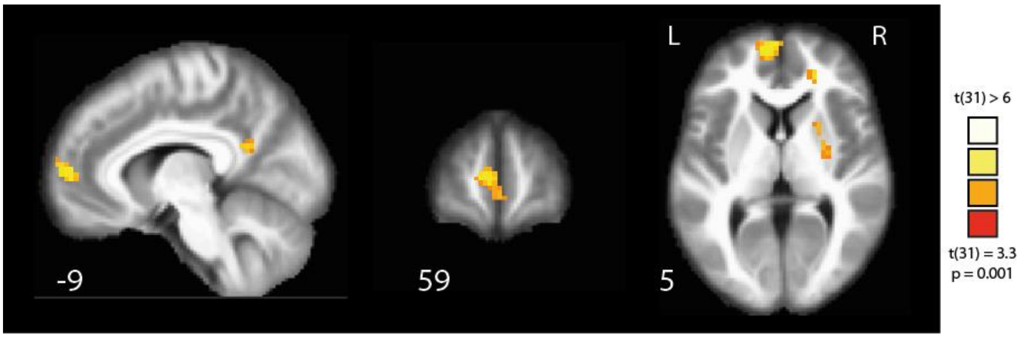

Supplementary Figure 7: Uncorrected Map of Relative Merit Prediction Errors effect during the outcome stage

In the uncorrected map from Figure 5A, at $p < 0.001$, it is possible to see relative merit prediction error effects in the Putamen and in the Precuneus, in addition to the MPFC activity which survived the cluster size correction.

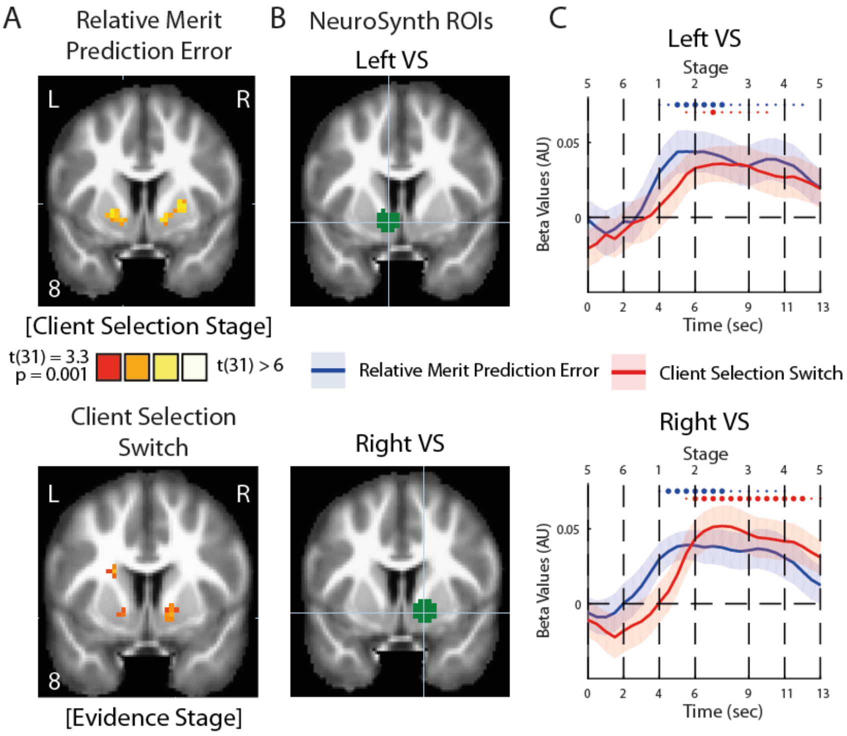

Supplementary Figure 8: Uncorrected Maps of Relative Merit Prediction Errors and Client Selection Switches in the Striatum

(A) In the uncorrected map from Figure 5B, at p < 0.001, and 5C, at p < 0.005, it is possible to see that the effects were presented both in the right VS, and to less extent, in the left VS. (B) ROIs of left and right VS defined by Neurosynth meta-analysis. (C) Time courses of the effects of Relative Merit prediction error (blue) and Cleint Selection Switches (red) from the left VS Neurosynth ROI (MNI coordinates [x,y,z]: 15,8,5]) and the right VS Neurosynth ROI (coordinates: [18,8,-10]). Time courses are presented across all stages of a trial, from the showdown stage (5) of a preceding trial to the showdown stage (5) of the current trial. Thick lines indicate mean effect size, the shaddows indicate the SEM, small dots indicate p < 0.05 in these time points, and big dots indicate p < 0.005.

Supplementary Tables

Model	Parameters					DIC
	$Bias$ (alpha)	γ	$\beta_{Selection}$	β_{Merit}	$\beta_{Interaction}$	
**Bias**	0.68±0.06					87.79±4.05
**Client**	0.72±0.06		-0.07±0.02			83.65±4.06
**Merit**	0.96±0.10	0.48±0.015		-0.36±0.07		92.07±4.1
**Mix**	0.74± 0.06	0.57±0.01	-0.08±0.03	0.006±0.02		84.75±4.15
**Interaction**	0.74±0.05	0.3±0.015	-0.08±0.02	0.003±0.02	-0.008±0.02	80.08±4.14
**Interaction Amplitude**	0.72±0.05	0.24±0.001	-0.08±0.02	0.02±0.02	0.009±0.01	83.48±4.15

Supplementary Table 1 - Model's parameters and fitting scores (Mean ± SEM)

				MNI Coordinates		
Sign	Region Name	Extent	t-value	x	y	z
Positive	R Angular Gyrus	22	4.51	42	-61	29

Supplementary Table 2– Activations in 'Chosen' > 'Ignored' client selection contrast during evidence stage, threshold at p < 0.001 (Relates to Figure 4) which survived cluster size FEW correction (p < 0.05). See full maps in NeuroVault: http://neurovault.org/collections/2204/

				MNI Coordinates		
Sign	Region Name	Extent	t-value	x	y	z
Positive	R Putamen	9	3.8	15	8	-1

Supplementary Table 3 – Modulation of activity during evidence stage by trial-by-trial Client Selection Switches, threshold at p < 0.001 (Relates to Figure 4C) which survived cluster size FEW correction (p < 0.05). See full maps in NeuroVault: http://neurovault.org/collections/2204/

				MNI Coordinates		
Sign	Region Name	Extent	t-value	x	y	z
Positive	L Superior Medial Gyrus	54	4.6	-6	62	8

Supplementary Table 4 – Modulation of activity during outcome stage by trial-by-trial Relative Merit PE, threshold at $p < 0.001$ (Relates to Figure 5A) which survived cluster size FEW correction ($p < 0.05$). See full maps in NeuroVault: http://neurovault.org/collections/2204/

				MNI Coordinates		
Sign	Region Name	Extent	t-value	x	y	z
Positive	R Caudate	60	5.47	15	11	-4

Supplementary Table 5 – Modulation of activity during appraisal stage by trial-by-trial Relative Merit PE, threshold at $p < 0.001$ (Relates to Figure 5B) which survived cluster size FEW correction ($p < 0.05$). See full maps in NeuroVault: http://neurovault.org/collections/2204/
